# The effect of a health extension program on improving water, sanitation, and hygiene practices in rural Ethiopia

**DOI:** 10.1186/s12913-023-09833-6

**Published:** 2023-08-07

**Authors:** Fikralem Alemu, Kasahun Eba, Zelalem Tazu Bonger, Ashrafedin Youya, Mulusew J. Gerbaba, Alula M. Teklu, Girmay Medhin

**Affiliations:** 1 MERQ Consultancy PLC, Addis Ababa, Ethiopia; 2 Department of Environmental Health Sciences and Technology, Jimma University, Jimma, Ethiopia; 3 Ohio State Global One Health, LLC, Addis Ababa, Ethiopia; 4 Ministry of Health, Addis Ababa, Ethiopia; 5 Department of Epidemiology, Jimma University, Jimma, Ethiopia; 6 Aklilu Lemma Institute of Pathobiology, Addis Ababa University, Addis Ababa, Ethiopia

**Keywords:** Health Extension Workers, WASH practices, Latrine ownership, Diarrhea, Hand-washing facility, Childhood diarrhea

## Abstract

**Background:**

To make basic primary health care services accessible, especially to the rural community, the government of Ethiopia launched the Health Extension Program (HEP) in 2004. Most of components of HEP are dedicated to hygiene and sanitation. Few studies have assessed the role of the Health Extension Program in improving water, hygiene, and sanitation (WASH) practices in Ethiopia. This study explored the role of health extension workers (HEWs) in influencing household water treatment practices, latrine ownership, latrine use and ownership, and the use of hand-washing facilities on the incidence of diarrheal diseases among the children under five years of age in rural Ethiopia.

**Methods:**

Using a cross sectional design, we conducted a national assessment that covered all nine regions of Ethiopia. We conducted face-to-face interviews among a sample of 6430 rural households using a structured questionnaire and an observation checklist to collect data from March 2018 to May 2019. Multilevel logistic regressions models were used to determine the relationships between the exposure of households to HEWs and WASH practice outcomes such as the use of water from an improved water source, household water treatment practices, availability of hand-washing and hand-washing with soap and water, availability of latrines, and use of latrines as well as the incidence of diarrheal diseases among children age 5 and younger. Our models were adjusted for covariates and confounders and *P*-values less than 5% were set to determine statistical significance.

**Results:**

We found that 72.7% of rural households had some type of latrine and 27.3% reported practicing open defecation. A total of 71.5% of rural households had access to drinking water from improved water sources, but only 9.4% reported practicing household water treatment. Exposure to HEWs was positively associated with household water treatment practices (AOR: 1.46; 95% CI = 1.01–2.10) and latrine availability (AOR: 1.44; 95% CI = 1.15–1.80). Among the households who were either visited by HEWs at their home or the that visited health posts to meet with the HEWs, being exposed to WASH health education by HEWs was significantly associated with the availability of a hand-washing facility (AOR: 5.14; 95% CI = 4.11–6.42) and latrine availability (AOR: 1.48; 95% CI = 1.10–2.01). However, we did not find a relationship between the incidence of diarrhea among children age 5 and under and exposure to HEWs (AOR: 2.09; 95% CI = 0.73– 6.62).

**Conclusion:**

Our results show a significant association between exposure to the Health Extension Program/ HEWs and improved household water treatment practices, latrine construction, and the availability of hand-washing facilities in rural Ethiopia, suggesting the need to strengthen efforts to change WASH behavior through the Heath Extension Program. On the other hand, further investigation is needed regarding the spillover effect of latrine use practices and the reduction of the incidence of diarrheal diseases.

## Background

Diarrheal disease is a serious public health threat that is closely associated with water, sanitation and hygiene (WASH) infrastructure and practices. Nearly 1.7 billion cases of childhood diarrheal disease occur worldwide, resulting in the death of 525, 000 children under the age of five each year [1], and repeated episodes are associated with growth impairment [2]. Unsafe water, inadequate sanitation, and poor hygiene were linked to 5–8% of diarrhea cases worldwide in 2012 [3]. Evidence shows that the provision of WASH services is associated with reduced diarrheal incidence. For instance, point-of-use water treatment with chlorine reduces the risk of diarrhea by 25–58% [4], improved sanitation can reduce diarrheal diseases by 32–37% [5], and hand-washing promotion reduces incidence of diarrhea by 42–47% [6], while combined water, sanitation, and hygiene interventions resulted in up to a 57% reduction in diarrheal incidence [7]. However, over 2 billion people worldwide lack access to water, more than one-third of the world’s population lacks basic sanitation, and only 19% of the worldwide population washes their hands with soap after using a sanitation facility or coming into contact with children’s excreta [8].

In Ethiopia, diarrhea is the cause of death for more than 73,341 children under the age of 5 annually [9]. Ethiopia has adopted the United Nation’s Sustainable Development Goal (SDG) 6, which is aimed at universal and equitable access to safe and affordable drinking water, sanitation, and hygiene for all by 2030 [10, 11]. Thus, the expansion of the WASH infrastructure, among other factors, has resulted in a remarkable decrease in diarrhea prevalence in Ethiopia. Diarrhea has decreased from 26% to 2000 to 18% in 2005, to 14% in 2011, and to 12% in 2016 [12]. However, diarrhea remains the second cause of morbidity and mortality among children under 5 in Ethiopia, after pneumonia. This is partly because Ethiopia has the poorest WASH infrastructure in the world. According to the World Health Organization (WHO) and the United Nations Children’s Fund (UNICEF) Joint Monitoring Program report, in 2017 only 14% of the Ethiopian population was using improved sanitation and 22% were practicing open defecation [13]. Less than 10% of the population reportedly had access to hand-washing facilities or practices that involved appropriate water treatment methods, with particularly low use in rural areas [13]. Studies in Ethiopia have found that latrine ownership and utilization and hand-washing practices are positively associated with positive attitudes and knowledge [14]. The study on hand-washing behavior from 51 Low and Middle Income Countries (LMICs) also reaffirmed that Ethiopia has the lowest proportion of households that have soap available in the hand-washing area [15]. Some studies in Ethiopia have also reported that interventions to change WASH behavior resulted in a decrease in childhood deaths [16, 17]. Several studies reported that socio demographic status such as increased education status [18], higher wealth index [19], and urban residency [20] were also associated with improved WASH practices.

Various strategies have been implemented to improve WASH practices. One study documented that community provision of improved sanitation facilities is required for effective WASH implementation [21]. Other effective strategies for WASH interventions include the complete separation of animal feces from the human environment [22] and the reduction of fecal contamination in spaces where young children crawl and play [23]. Other strategies include continuous and convenient access to uncontaminated water [24], various behavioral change modalities [25], and new technologies for delivering WASH services [26].

Ethiopia is a country characterized by pastoral and agrarian societies. Pastoral societies can be found in Afar, Ethiopian Somali, some parts of Oromia, and some parts of the Southern Nations, Nationalities and Peoples’ Region (SNNPR). These pastoral settlements contain small, scattered, and often nomadic populations, which makes it more challenging to provide basic public services such as clean water delivery. HEWs were introduced into the health system in agrarian areas in 2004 and were scaled up to serve pastoral areas in 2006 [27]. To improve disparities in access to health services, including WASH, between regions and different settings, the government established context-specific implementation models for rural (agrarian), urban, and pastoral societies. So far 38,000 HEWs have been trained and deployed, 87% of whom were deployed to rural (agrarian), 10% to urban, and 3% to pastoral societies [28].

Ethiopia has put a great deal of effort into meeting SDGs, and this includes the launching of theHealth Extension Program (HEP) [29]. As part of implementing HEP, the government of Ethiopia deployed trained community-based health workers, namely, Health Extension Workers (HEWs). HEWs provide health in-home education (12.8% time), basic curative, promotive, and preventive services at their health posts (25% time), including the promotion of household toilets, hand-washing with soap (or ash) at critical times, and safe drinking water handling and treatment home, and building relationships with the community and mobilizing the community (14% time) [30]. A network of women’s groups that comprises 25 to 30 households was organized, with one woman out of every five households becoming a Women’s Development Army leader who is responsible for promoting the health of five neighboring households under the supervision of HEWs [31].

However, evidence is scare regarding the contribution of Ethiopia’s HEP in improving access to and the use of WASH services as well as the reduction of diarrhea incidence. Nor has the effectiveness of the HEP in reducing childhood diarrheal disease been well studied. Thus, the aim of this study is to evaluate the access to improved drinking water, proper hygiene, and proper sanitation and to explore the association between improved WASH practices and exposure to information/communication by Health Extension Workers.

## Methods

### Study context

Ethiopia is the second most populated country in Africa, with an estimated population of 105 million in 2017 of which 84% reside in rural settings [32]. Administratively, Ethiopia is divided hierarchically into regions, zones, woredas (districts) and kebeles (wards), with the kebele being the smallest administrative unit of the government. During data collection there were 11 administrative structures: 9 regional states (Tigray, Afar, Amhara, Oromiya, Somali, Benishangul-Gumuz, Southern Nations Nationalities and Peoples (SNNP), Gambela, and Harari), and 2 city administrations (Addis Ababa and Dire Dawa). The administrative structure for decision-making for HEP implementation is shared between the Federal Ministry of Health, the Regional Health Bureaus, the Woreda Health Office, and the kebele. HEWs are situated at the kebele level in Health Posts (HPs) and serve 3000–5000 people per kebele [33].

#### Study design

The analysis for this mixed method study was based on data that were collected for a national health extension program assessment from March 2018 to May 2019. This national HEP assessment was broad and included selected kebeles (the lower administration unit) of all the regions in the country.

### Sample size and sampling procedures

Details on sample size estimation and the sampling procedure for the national survey are reported elsewhere [34]. Considering the woredas as the primary sampling unit, we randomly selected 62 woredas from the 9 Ethiopian regions. A random number generator was used to randomly select woredas. Three kebeles from each woreda were randomly selected (n = 186). At each kebele, we randomly selected 34 households and eligible women to take part in an interview and we interviewed one woman in the household. Among the 7122 rural households that participated in the study, we excluded 692 Women Development Army (WDA) participants from our analysis because the data collected from the WDA to address a different objective which is beyond the scope of this paper. Thus, a total of 6430 sampled households were included in our analysis. Moreover, urban areas (Addis Ababa city Administration and Dire Dawa City administration) were surveyed in a parallel survey but excluded from our analysis.

### Data collection method and procedure

We collected data using face-to-face interviews with women of the households and conducted observations. We used two standardized questionnaires (the Household Questionnaire and the Woman’s questionnaire) adapted from the standard DHS program for Ethiopia [35]. The Household Questionnaire was used to collect information about the household members’ demographics, source of water, type of toilet facilities, materials used for the floor of the dwelling unit, and ownership of various durable goods. The Woman’s Questionnaire was used to collect information on the availability of toilet and hand-washing facilities, and the household members’ WASH practices, which included hand-washing, point of use (POU), water treatment, face and body washing, clothes washing, shoe wearing, house cleanliness, disposal of solid and liquid waste, and the keeping of livestock separate from the living room. Participants were also asked if they had received health education and then engaged in discussions with the HEW on various topics such as personal hygiene, latrine construction and use, household water treatment, and food hygiene during HEW’s visit to their house during the year prior to the time of data collection. In addition to the interview, we observed the households using a checklist. The checklist contained the same components as the Household Questionnaire and the Women’s Questionnaire, including the availability and type of toilet facilities, availability of hand-washing facilities and their functionality, and the presence of soap and water. During the preparation for the original assessment for which this data was extracted, we translated the questionnaires and checklist from English into six local languages, namely, Amharic, Tigrigna, Somale, Afar, Afaan Oromoo, and Agnuak, and back-translated them to English to ensure consistency and the accuracy of each item. The translators had experience translating similar questions and were trained in the use of appropriate terminology. We conducted a pretest with 5% of the sample in Addis Ababa to test the survey instrument and data collectors’ proficiency and made any necessary modifications.

The field data were collected using computer assisted software ODK (open data code). We recruited data collectors who had at least a BSC degree and supervisors who had a master’s degree. The data collectors provided with ten days of training on interviews, survey questions, and sampling procedure. Supervisors were responsible for monitoring the data collection procedures and crosschecking the quality of the collected questionnaires on a daily basis. We then exported the data to Software for Statistics and Data Science (STATA) software version 16 for cleaning and further analysis.

### Measurements

#### Outcomes of interest

We used six outcome variables: status of drinking water use from improved water sources (i.e., sources that are likely to be protected from outside contamination, particularly from fecal matter) (binary variable coded 1 for yes and 0 for no); household water treatment practice (binary variable coded 1 if household practiced water treatment and 0 if it did not ); availability of hand-washing facility (coded 1 for yes 0 for no); availability of latrine (coded 1 when latrine was available and 0 if unavailable); and use of latrine (coded 1 when they said they used it and 0 if not). Data related to diarrhea were collected from the mothers/primary care takers using an interviewer-administered questionnaire. A 2-week recall method was used to assess the prevalence of diarrhea. Diarrheal incidence (the passage of three or more loose or liquid stools within a 24-hour period) in the last 2 weeks in one youngest under five year old childin a family was recorded (which was coded 1 for yes and 0 for no).

### Exposure variables

We used two main exposure variables, namely, (a) having an exposure to HEWs while the household visiting health post or the HEW visited the HH for any reason within a one-month period prior to data collection period (coded 1 = yes if the household (HH) was exposed to HEWs and 0 = no if otherwise), and (b) having exposure to a discussion with the HEWs about WASH-related topics such as excreta disposal, solid and liquid waste disposal, water supply and safety measures, food hygiene and safety measures, healthy home environment, control of insects and rodents, and personal hygiene (yes coded 1 and no coded 0).

### Confounding and other covariates

Many studies have identified associations between poor WASH practices and sociodemographic and economic factors [36, 37]. Thus, our analysis on the association between exposure to HEP with WASH practices was adjusted for the sociodemographic and economic status of the study participants, namely, age, sex, education level, family size, residential area (agrarian or pastoral areas), and the wealth index of the household.

### Operational definitions

*Improved drinking water sources*: water sources that are designed to protect against contamination, which include piped water, boreholes or tube wells, protected dug wells, protected springs, and packaged or delivered water [38].

*Unimproved sources*: water sources from unprotected wells, unprotected springs, or surface water.

*Improved sanitation*: sanitation facilities that are designed to hygienically separate excreta from human contact such as flush/pour-flush toilets, ventilated improved pit latrines, composting toilets, and pit latrines that have a slab or platform.

*Unimproved sanitation*: sanitation facilities that include pit latrines without a slab or platform, hanging latrines, and bucket latrines.

*Family size*: number of adults and children.

### Data analysis

We reported weighted results to take into account the disproportionate allocation of the study participants to different regions and the use of different probability sampling in the selection of study participants as well as to enable the findings to be generalized for the whole country. We first computed descriptive statistics and presented them in tables. We then used a weighted multilevel logistic regression model to address the stratified three-stage sampling design to identify study participants. To take into account the dependency/correlation that might arise within each cluster/level, we introduced two random effects in the model. As a first step, a pairwise multilevel logistic regression using each of the outcome variables was applied to determine unadjusted effects for each of the exposure variables including the socio demographic variables. We subsequently included the variables with a *P*-value < 0.05 in the final multilevel regression model to assess the independent effect after controlling for other variables. We evaluated the importance of each level/grouping by measuring the degree of clustering within each level using the intra-class correlation coefficient (ICC). Both crude and adjusted odd ratios with corresponding 95% confidence intervals were reported as measures of associations. A *P*-value of less than 0.05 was considered a statistically significant association.

## Results

We analyzed data collected from 6430 women of which 95.6% were agrarian. Most participants (85%) were married and 75% had a family size of 4 or more, 38% were under 30 years of age, with the mean age being 38.8 (SD = 13.7). The majority (71%) of participants were illiterate or had only primary education (25%), and 34.5% were in the lower wealth group (Table 1).

**Table 1:** Sociodemographic characteristics of the study participants

Characteristics	Category	Number of women (unweighted)	Percentage (weighted)
Residence	Pastoralist	2009	4.38
	Agrarian	4421	95.62
Wealth	Low	2634	36.44
	Middle	1278	20.42
	High	2518	43.14
Marital status	Currently Married	5162	84.9
	Single/Divorced/widowed/separated	1268	15.1
Education status	No education	4819	70.75
	Primary education	1329	25.22
	Secondary education and above	282	4.04
Age category	< 30	2755	31.02
	30–50	2551	44.42
	50+	1124	24.56
Family size	<=3	1739	25.44
	4–5	2206	35.69
	6+	2485	38.87

### Access to WASH

We found that 72.7% of the households had at least one latrine type, whereas 27.3% reported practicing open defecation by both adults and children at the time of data collection. Out of the latrine owners, 29.2% had improved facilities (23.3% had pit latrines with slabs and 5.5% had ventilated improved latrines), while 70.8% had unimproved latrines. Among those who had any type of latrine, 98.5% reported latrine usage by most of the family members.

In addition, 71.5% of the women reported that they were drinking water from improved water sources. Only 9.4% of the households reported practicing water treatment and of these, 15% practiced boiling water, 73% added chlorine to the water, 8% used a water filter (ceramic/sand/comp), and 4% used other water treatment methods. This study also revealed that only 8.3% of the HHs had a hand-washing facility near the latrine, 6% had a latrine with water, and 4% had a latrine with soap and water available near the hand-washing facility that was confirmed through observation of the HHs. On the other hand, during interview with women on self-reported hand-washing practices at critical times, 93.4% reported washing their hands before feeding, 80.6% reported washing their hands after feeding, 70.7% reported washing their hands before food preparation, 16% reported washing their hands before breast-feeding, and 61.2% reported washing their hands after using the toilet.

### Associations of WASH practice and exposure to health education through HEWs

Table 2 summarizes the results from multilevel logistics regression, which shows the associations between exposure of households to health education delivered by HEWs and four WASH practices. Accordingly, exposure to the health education delivered by HEWS is positively and significantly associated with household water treatment practices (AOR: 1.46; 95% CI = 1.01–2.10) and latrine availability (AOR: 1.44; 95% CI = 1.15–1.80).

**Table 2:** Results from multilevel logistics regression analysis showing the association of WASH practices with exposure to HEWs

Outcomes	Household had exposure to HEWs		AOR (95%CI)*
	Yes, %	No, %	
Water treatment practice	9.40	6.02	1.46 (1.01, 2.10)
Availability of hand-washing facility near latrine	3.02	1.56	1.48 (0.52, 4.23)
Latrine use practice	98.74	98.23	1.29 (0.31, 5.49)
Latrine availability	74.32	62.74	1.44(1.15, 1.80)

*Adjusted for residence, age, wealth, education, marital status, and family size

Table 3 summarizes the results from multilevel logistic regression analysis, where the outcomes are status of WASH practices and exposures to HEWs and having discussions about relevant WASH topics during most recent exposure to HEWs. Among those who were either visited by HEWs at their home or the households who visited health posts to meet with the HEWs, having exposure to health education on relevant WASH topics by HEWs had a positive significant association with the availability of a hand-washing facility (AOR: 5.14; 95% CI = 4.11–6.42) and latrine availability (AOR: 1.48; 95% CI = 1.10– 2.01). However, latrine use practice had a negative association with having exposure to health education (AOR: 0.62; 95% CI = 0.44–0.89). There was no significant association between exposure to HEWs and household water treatment practice (AOR 1.24; 95% CI = 0.77–2.02).

**Table 3:** Results from the multilevel logistics regression analysis showing the association of exposure to a discussion on related WASH topic with the four outcomes

Outcomes	Exposure to HEW	Category	AOR (95% CI) *
Water treatment practice	Water supply and food hygiene	Not discussed	Ref
		Discussed	1.24(0.77, 2.02)
Latrine use practice	Latrine construction & Utilization	Not discussed	Ref.
		Discussed	0.62(0.44, 0.89)
Availability of hand-washing facility	Personal hygiene	Not discussed	Ref.
		Discussed	5.14(4.11, 6.42)
Latrine availability	Latrine construction & Utilization	Not discussed	Ref.
		Discussed	1.48(1.10, 2.01)

*Adjusted for residence, age, wealth, education, marital status, and family size

### Relationships of incidence of diarrhea with WASH practices, exposure to HEWs and Socio demographic variables

Table 4 summarizes multivariable logistics regression analysis of the relationship between the incidence of diarrhea in the past 2 weeks among the under-5 children and exposure to HEW, adjusted for WASH practices and sociodemographic characteristics. We found no significant association between incidence of diarrhea among the under-5 children and exposure to HEW (AOR: 2.09; 95% CI = 0.73 − 6.62). The incidence of diarrhea among the children under 5 is more likely to be lower among households who were practicing household water treatment (AOR: 2.87; 95% CI = 1.38 − 5.95), whereas there was no significant association between incidence of diarrhea among under-5 children and use of latrine (AOR: 3.04; 95% CI = 0.26– 34.95), having a hand-washing facility (AOR: 0.69; 95% CI = 0.15–3.28) or sociodemographic variables that include age, education status, wealth, marital status, and family size.

**Table 4:** Results of the multivariate logistics regression analysis showing the association between exposure to HEP with past two weeks’ incidence of diarrhea among under-five children

Factors	Categories	AOR (95% CI)
Visit either	Yes (ref: No)	2.09(0.73, 6.62)
Water treatment practice	Yes (ref: No)	2.87(1.38, 5.95)
improved water source	Yes (ref: No)	0.72(0.31, 1.67)
Latrine use practice	Yes (ref: No)	3.04(0.26, 34.95)
Availability of hand-washing facility near latrine	Yes (ref: No)	0.69(0.15, 3.28)
Agrarian/pastoralist	Pastoralists (Ref: agrarian)	0.79(0.31, 2.03)
Wealth	Middle (Ref: Low)	1.02(0.58, 1.81)
	High (Ref: Low)	1.06(0.54, 2.07)
Age	30–50(Ref: <30)	0.58(0.34, 1.01)
Formal Education (Ref: No education)	Primary	0.79(0.35, 1.75)
	Secondary and above	0.43(0.12, 1.50)
Marital Status	Currently Married	0.35(0.09, 1.39)
Family Size (Ref: <3)	3–5	0.94(0.35, 2.54)
	6+	1.09(0.42, 2.81)

## Discussion

This study investigated the association between safe water use, sanitation, and hygiene practice, and the incidence of childhood diarrhea and the exposure to information and communication to the health extension program in rural Ethiopia. Our findings demonstrate that most of the households had some type of latrine and had access to drinking water from an improved water source. Moreover, exposure to the Health Extension Workers was associated with a household’s increased water treatment practice and latrine availability as well as the availability of a hand-washing facility. This finding is in line with the results of previous studies that reported the positive effects of WASH education on household latrine availability and water treatment [39–41]. Similarly, studies conducted in different parts of Ethiopia showed that WASH education was significantly associated with latrine ownership in households [18, 42]. Other studies in different parts of the world have also reported a positive impact of WASH promotion on improved WASH practices [39–41, 43–45]. This indicates that hygiene promotion and health education in rural settings such as in Ethiopia might contribute to the improvement of WASH practices such as household latrine ownership as well as water treatment and safe storage practices [46]. Thus, the health extension program in Ethiopia needs to be revised so that a comprehensive package of WASH interventions can be implemented with the support of HEWs.

On the other hand, latrine use was negatively associated with exposure to health education by health extension workers. Previous studies reported a mix of positive and negative associations. Evidence has also shown that an increase in knowledge may not necessarily result in improved practice [47] and that people may have sufficient knowledge about the risk of open defecation causing diarrhea but providing them health awareness may not improve latrine use practice. Previous studies have documented that people may prefer to defecate openly due to sociodemographic, behavioral, and environmental factors [48, 49]. Moreover, latrine cleanliness, water availability, latrine maintenance conditions, the presence of an under-5 child and personal beliefs were associated with lower latrine use [49–51]. In addition, the HEWs visit to homes might not target WASH activities as they have a number of health extension packages to be covered. Thus, to increase the use of latrines and reduce the prevalence of diarrhea, manuals need to be prepared that cover WASH activities during home visits. Similarly, several hygiene and sanitation behavior change interventions that were focused on educating people about the risks of disease failed to bring sustainable behavior change, which suggests that interventions on sanitation must take into account different approaches to address other behavioral attributes, including social norms, beliefs, and attitudes [52]. The Health Extension Program in Ethiopia needs to identify and address the behavioral barriers of latrine use to improve the effectiveness of WASH interventions.

In this study, the majority of the households (73%) owned some type of latrine, while 27.3% reported practicing open defecation, which is in line with a previous Ethiopian study that documented the same level of latrine ownership [53]. However, the latrine ownership reported in our study is lower than the one reported in North West Ethiopia, where the majority (89.7% and 92.8%) of both open defecation-free and open defecation kebeles had a private latrine, respectively [54] and in eastern Ethiopia, where 89% have latrines [55]. On the other hand, the latrine coverage in this study is higher than the one reported in rural villages of the country [56, 57]. In this study, the majority (70.8%) of latrine owners had unimproved latrines, which is in line with a previous study in Ethiopia [18]. Thus, the Health Extension Program needs to increase efforts to promote latrine ownership and utilization in order to prevent diarrhea in Ethiopia.

This study found no significant association between the incidence of diarrheal disease and exposure to health workers or discussion about specific WASH topics. Conversely, previous studies documented that WASH educational intervention significantly reduced the incidence of childhood diarrhea [58, 59]. This discrepancy might be due to WASH topics not being covered during exposure to HEWs or that the specific WASH topics covered did not target diarrhea prevention. In addition, there might have been behavioral resistance from the community and this warrants further investigation.

The incidence of diarrheal disease among under-5 children in the household was more likely to be lower among households who were practicing household water treatment. The water treatment practice shown in this study is lower than that of a study conducted in southern Ethiopia, which reported 44.1% household water treatment practices [60] Another study demonstrated that household water treatment was key to meaningful reductions in diarrhea [61]. The results of our study showed that there was no significant association between incidence of diarrhea among under-5 children and the availability of hand-washing facilities. This finding differs from other studies, which documented a strong association between an improved hand-washing practice and reduced incidence of diarrhea [62–67]. The reason could be that our analysis was based on the availability of hand-washing facilities rather than the practice and that the availability of hand-washing facilities does not guarantee that people will improve their hand-washing practices. This study has also shown that there was no positive association between diarrheal incidence and latrine availability or latrine use. Similar to this finding, the results of some studies on the determinants of childhood diarrhea in Ethiopia reported no significant association between the availability of any type of latrine with childhood diarrhea [68–71]. On the other hand, other studies have indicated that the absence of any type of latrine was positively associated with childhood diarrhea [72, 73]. Several studies have reported that despite the availability of latrine, lack of cleanliness of a latrine might contaminate the surrounding area and thus cause infection for diarrhea [74, 75]. Hence, we recommend further investigation on how the quality of latrines affect their usage and its impact on decreasing childhood diarrhea.

### Strengths and limitations

The key strength of this study is that it was conducted on a large national scale that is representative of all regions of Ethiopia. Although this study has generated programmatically useful information, it is not without limitations. Because of the cross-sectional study design used to collect the data, having only one-time post-intervention data made it difficult to establish a causal relationship between the exposure and the observed outcomes. The analysis on household water treatment, hand-washing at critical times, and latrine use were based on the participants’ self-reported responses, which might reduce the level of confidence concerning the strength of our conclusion.

## Conclusion

Our findings show a significant positive association between exposure to Health Extension Program/HEWs and improved household water treatment practices, latrine construction, and the availability of hand-washing facilities in rural Ethiopia. This finding suggests the need to strengthen efforts to change WASH behavior as part of the Health Extension Program. On the other hand, latrine use practice was negatively associated with exposure to health education, indicating that there might be behavioral barriers to latrine use. The lack of association between exposure to HEW and childhood diarrheal disease suggests that HEW interventions alone may not be sufficient to decrease childhood diarrhea and that more measures are called for.

## Data Availability

All the necessary data were analyzed and included in this manuscript. For further need, data can be obtained from the corresponding author upon request.

## Abbreviations

AOR: Adjusted Odds Ration
COR: Crude Odds Ration
HEW: Health Extension Workers
HEP: Health Extension Program
HH: Households
LMICs: Low- and Middle-Income Countries
POU: Point of Use
SD: Standard Deviation
SNNP: Southern Nations and Nationalities
UNICEF: United Nations Children’s Fund
WASH: Water, Sanitation and Hygiene
